# Comparative analysis of individual chromosome involvement in micronuclei induced by mitomycin C and bleomycin in human leukocytes

**DOI:** 10.1186/s13039-016-0258-4

**Published:** 2016-06-21

**Authors:** Galina Hovhannisyan, Rouben Aroutiounian, Nelly Babayan, Tigran Harutyunyan, Thomas Liehr

**Affiliations:** Department of Genetics and Cytology, Faculty of Biology, Yerevan State University, 1 Alex Manoogian, 0025 Yerevan, Armenia; Institute of Molecular Biology, National Academy of Sciences, 7 Hasratyan, 0014 Yerevan, Armenia; Jena University Hospital, Friedrich Schiller University, Institute of Human Genetics, Kollegiengasse 10, D-07743 Jena, Germany

**Keywords:** Micronuclei, Chromosome, FISH, Mitomycin C, Bleomycin, Human leukocytes

## Abstract

**Background:**

Micronucleus (MN) assay is a well standardized approach for evaluation of clastogenic/aneugenic effects of mutagens. Fluorescence in situ hybridization (FISH) is successfully used to characterize the chromosomal content of MN. However, the relationships between nuclear positioning, length, and gene density of individual chromosomes and their involvement in MN induced by different mutagens have not been clearly defined.

**Results:**

Chromosomal content of MN was characterized in human leukocytes treated with mitomycin C (MMC) and bleomycin (BLM) by FISH using centromeric (cep) and whole-chromosome painting (wcp) probes. Involvement of chromosomes 8, 15 and 20 in MMC-induced and chromosomes 1, 9 and 16 in BLM-induced MN was studied, and correlated with chromosome size, gene density and interphase position. The results obtained were analyzed together with previous own data on the frequencies of inclusion of chromosomes 3, 4, 6, 7, 9, 16, 17, 18, and X in MMC-induced MN. It could be shown that MMC- and BLM-induced MN could contain material derived from all chromosomes investigated. Involvement of whole chromosomes 8, 15 and 20 in MMC-induced MN negatively correlated with gene density; however, analysis together with earlier studied chromosomes did not confirm this correlation. Inclusion of chromosomes 8, 15 and 20 in MMC-induced MN does not depend on their size and interphase position; the same result was found for the twelve overall analyzed chromosomes. In BLM-treated cells significant correlation between frequencies of involvement of chromosomes 1, 9 and 16 in MN and their size was found.

**Conclusions:**

Our results clearly revealed that BLM differs from MMC with respect to the distribution of induced chromosome damage and MN formation. Thus, DNA-damaging agents with diverse mechanism of action induce qualitatively different MN with regard to their chromosomal composition. Also this study demonstrates the utility of combined sequential application of cep and wcp probes for efficient detection of MN chromosomal content in terms of centric and acentric fragments.

## Background

In classical cytogenetics chromosomes are studied directly by observing and counting aberrations in metaphases. The micronucleus (MN) assay is an alternative and simpler approach to assess chromosomal damage. MN are small, extranuclear bodies that originate from acentric fragments or whole chromosomes which usually get lost from the cell nucleus during mitosis [1, 2]. The occurrence of MN is considered to be a good indicator of clastogenic and/or aneugenic effects. MN can only be expressed in cells which completed nuclear division. Elaboration of the cytokinesis-block micronucleus (CBMN) assay based on the chemical blocking of cell division with cytochalasin B has made it possible to recognize once-divided cells by their binucleated appearance. This modification significantly increased efficiency of the MN analysis. CBMN assay is one of the most commonly used methods in genotoxicity testing [3] and human biomonitoring [2].

However CBMN test does not allow determining chromosomal composition of MN. Fluorescence in situ hybridization (FISH) is widely used to localize chromosome damage in genetic toxicology [4] and to detect genetic aberrations of medical significance [5]. Fluorescent DNA probes that bind defined genomic sequences are successfully applied to characterize the chromosomal content of spontaneous and mutagen-induced human MN. X and Y chromosomes have been shown to predominate in *spontaneous* MN [6, 7]. Different frequency of the involvement of various autosomes in *spontaneous* MN was demonstrated using spectral karyotyping and confirmed by FISH, as well [8]. Among autosomes, the fragments from chromosome 9 are the most prevalent in *spontaneous* MN and fragments from chromosomes 1, 9 or 16 are the most commonly found in MN induced in vitro by mitomycin C (MMC), 5-azacytidine and idoxuridine as a result of breakage in heterochromatic sites of these chromosomes [7]. Higher frequencies of chromosome 8, than chromosome 7 were detected in MN in human lymphocytes treated with the benzene metabolite, 1,2,4-benzenetriol [9]. Random involvement of chromosomes in radiation-induced MN, depending on the DNA content, was shown by [10]. Other studies demonstrated both random and non-random incorporation of DNA from different chromosomes in radiation-induced MN, larger chromosomes being usually overrepresented in the MN content [11, 12].

Chromosomes can be thought to be incorporated into MN depending on their size and/or gene density [13], as well as the general nuclear chromatin organization [14, 15], and/or lethality of chromosomal (partial) loss for the cells [9]. However, despite existing information the data on involvement of various types of chromosomes and chromosomal fragments in mutagens-induced MN is still limited, even though further characterization of MN contents is crucial for understanding and accurate application of the MN assay.

Earlier we analyzed the chromosomal composition of MN induced by MMC in human peripheral blood leukocytes [16, 17]. For chromosomes 3, 4, 6, 7, 9, 16, 17, 18 and X in MMC-induced MN no correlation for their nuclear positioning, length, and/or gene density could be found. However, chromosomes 9 and 16 were involved in MN-formation more frequently than expected according to DNA-content [17]. The aim of this study was to expand our earlier data by analyzing the involvement of chromosomes 8, 15 and 20 in MMC-induced MN. Besides, chromosomes 1, 9 and 16, being the most sensitive ones toward MMC-treatment [15, 17] were tested in bleomycin (BLM)-induced MN. As in previous studies sequencial FISH using centromeric (cep) and whole-chromosome painting (wcp) probes was performed.

## Results

### Number of wcp and cep signals in MMC- and BLM-induced MN

**Fig. 1:**
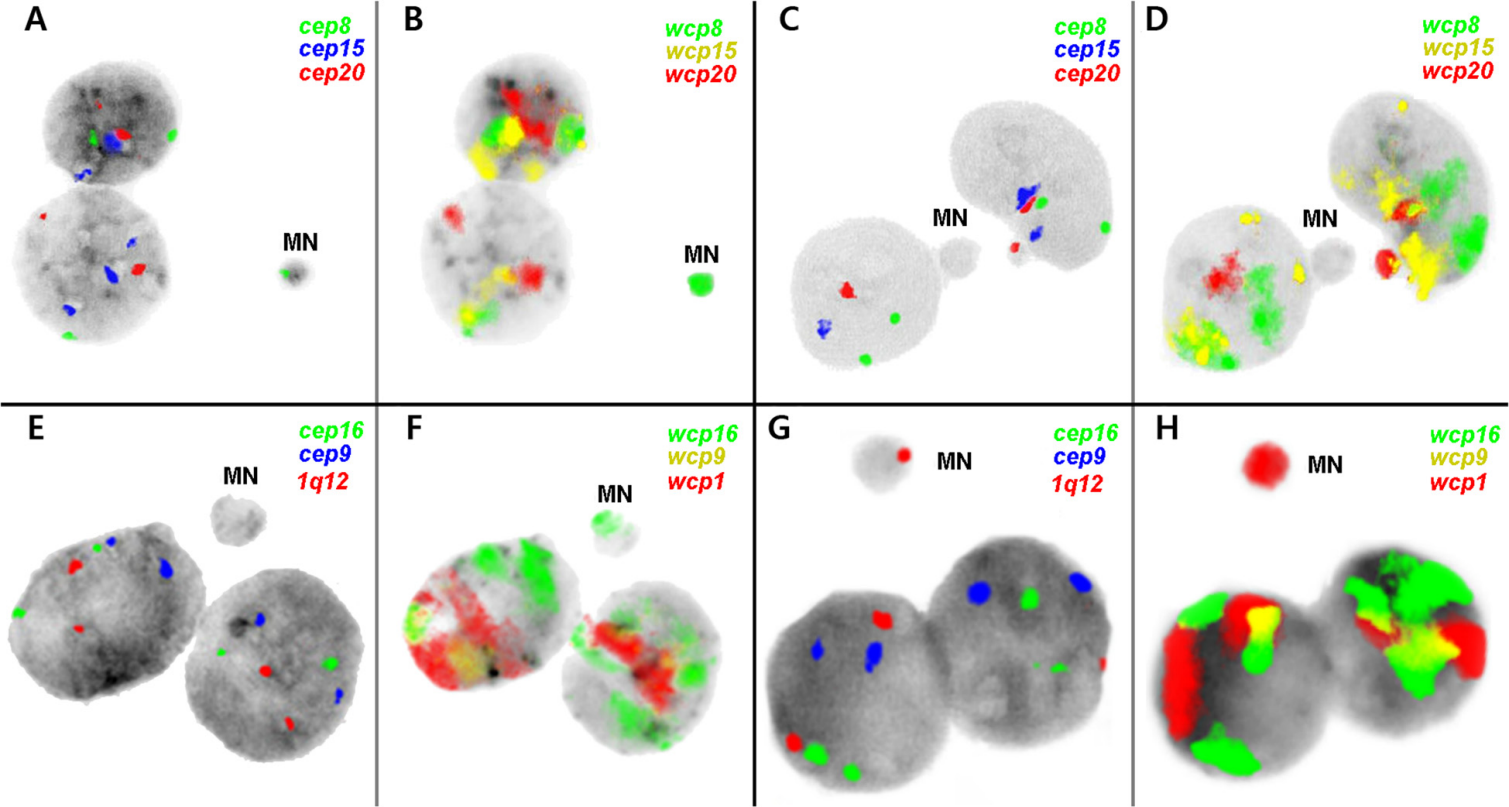
Chromosomal content of micronuclei (MN) in binucleated human leukocytes. The MN in mitomycin C- and bleomycin-treated human leukocytes were sequentially analyzed by three-color FISH using centromeric probes (cep) and whole chromosome paints (wcp). Mitomycin C-induced MN containing centromeric (**a**) and euchromatin-derived (**b**) material of chromosome 8. Mitomycin C-induced MN with centromeric (**c**) and euchromatin-derived (**d**) material of chromosome 20. Bleomycin-induced MN containing only euchromatic DNA derived from chromosome 16 (**e** and **f**). Bleomycin-induced MN with signal for 1q12 (**g**) and euchromatin-derived material of chromosome 1 (**h**)

In the present study we have identified the chromosomes and chromosomal fragments involved in MN induced by MMC and BLM. Chromosomal content of MN is summarized in Table 1. Representative pictures of MMC- and BLM-induced MN are shown in Fig. 1. According to the results obtained 1.6–2.8 times more MN containing wcp than cep signals were detected in MMC-treated cells and 3.5–8.14 times more in cells treated with BLM. Thus, a clear predominance of wcp over cep signals, especially in BLM-treated cells was revealed, highlighting a certain rate of acentric fragments included in MN.

**Table 1:** Frequencies of MMC- and BLM-induced MN with centromeric and whole-chromosome painting signals

Chromosome number	Total number of MN	Number (%) of MN with cep signals	Number (%) of MN with wcp signals
	MMC-induced MN		
8	1,262	19 (1.51)	31 (2.46)
15	1,262	16 (1.27)	32 (2.54)
20	1,262	7 (0.55)	16 (1.27)
	BLM-induced MN		
1	1,387	14 (1.01)	114 (8.22)
9	1,387	10 (0.72)	65 (4.69)
16	1,387	10 (0.72)	35 (2.52)

*cep* centromeric, *MMC* mitomycin C, *BLM* bleomycin, *MN* micronuclei, *wcp* whole-chromosome painting

Implementation of cep and wcp probes for the studied chromosomes revealed frequencies of involvement of chromosomes 8, 15 and 20 in MMC-induced MN and chromosomes 1, 9 and 16 in BLM-induced MN in human leukocytes

### Dependence of chromosomes inclusion in MN on their length, gene density and original localization in nucleus

The original chromosomal localization in the interphase nucleus is based on data of [18] for human lymphocytes and [19] for human sperm since for most of the chromosomes, the distribution of the territories seems to be similar in sperm and lymphocytes apart from the acrocentric chromosomes [20]. Data on intermediate localization of only acrocentric chromosome 15 in human fibroblasts [21] are in line with data for human sperm. The data displaying positions of chromosomes investigated, their length and gene density [22] are presented in Table 2.

**Table 2:** Original localization in the interphase nucleus, length, and gene density of the studied chromosomes

Chromosome number	Chromosomal distribution within the interphase nuclei (ratio of central to peripheral fractions)^a^	Chromosome length^b^ (Mb)	Gene density (genes per Mb)^b^
1	0.43	249.3	7.86
8	0.36	146.4	4.36
9	0.50	141.2	5.30
15	0.88	102.5	5.33
16	1.14	90.4	8.67
20	0.43	63.0	8.22

^a^Data from [19]

^b^Data from [22]

The frequency of cep- and wcp-positive MN in MMC-treated cells did not correlate with the length of chromosomes (*r* = 0.952 and *r* = 0.819, respectively) or their localization in the interphase nucleus (*r* = 0.126 and *r* = 0.413, respectively) (*p* > 0.05 for all cases, Table 3). Strong negative correlation was found between number of cep-positive MN and gene density (*r* = −1.000; *p* < 0.01).

**Table 3:** Correlations between MMC-induced cep- and wcp-positive MN and chromosomes nuclear localization, length and gene density

	Chromosome localization	Chromosome length	Gene density
MN with cep	0.126	0.952	−1.000*
MN with wcp	0.413	0.819	−0.955

Statistically significant negative correlations are indicated at *p < 0.01

Analysis of Pearson correlation (r) revealed statistically significant negative correlation between MMC-induced MN with cep and gene density (*p < 0.01)

The frequencies of wcp signals of chromosomes 1, 9 and 16 in MN in BLM-treated cells are proportional to chromosomes length (*r* = −0.998; *p* < 0.05) (Table 4). At the same time, micronucleation of whole chromosomes and their fragments was independent of their localization in nucleus (*r* = −0.609 and *r* = −0.020 for cep and wcp respectively; *p* > 0.05) and gene density (*r* = −0.356 and *r* = −0.112 for cep and wcp respectively; *p* > 0.05).

**Table 4:** Correlations between BLM-induced cep- and wcp-positive MN and chromosomes nuclear localization, length and gene density

	Chromosome localization	Chromosome length	Gene density
MN with cep	−0.609	0.950	0.287
MN with wcp	−0.863	0.998*	−0.095

Statistically significant positive correlations are indicated at *p < 0.05

Analysis of Pearson correlation (r) revealed statistically significant positive correlation between BLM-induced MN with wcp and chromosome length (*p < 0.05)

### Involvement of individual chromosomes in MN

The micronucleation of each chromosome was analyzed to disclose chromosome-specific effects of mutagens studied. To analyze a potential correlation for inclusion of specific chromosomal material into MN depending *on chromosomal size* the obtained numbers of wcp-positive MN were compared with the expected number, calculated on the basis of DNA content. Chromosomes 8, 15 and 20 were involved in MMC-induced MN less often than expected, with a significant value only for chromosome 8 (*p* < 0.01). No significant difference was observed between obtained and expected involvement of chromosomes 1, 9 and 16 in BLM-induced MN (*p* > 0.05), indicating for a proportional distribution of cytogenetic damage based on DNA content (Table 5).

**Table 5:** Observed and expected frequencies of involvement of the studied chromosomes in MMC– and BLM-induced MN

Chromosome number	Observed (wcp + MN)	Expected (wcp + MN)	Observed (wcp - MN)	Expected (wcp - MN)	*χ* ^2^	Total MN
	MMC-induced MN					
8	31*	60	1,231	1,202	14.715	1,262
15	32	42	1,230	1,220	2.461	1,262
20	16	26	1,246	1,236	3.926	1,262
	BLM-induced MN					
1	114	112	1,273	1,375	0.037	1,387
9	65	63	1,222	1,324	0.066	1,387
16	35	41	1,252	1,346	1.410	1,387

wcp + MN, wcp signal-positive MN; wcp- MN, wcp signal-negative MN

*p < 0.01 when compared with expected value (*χ* ^2^ test)

Comparison of observed and expected frequencies of micronucleation using *χ* ^2^ test revealed that chromosome 8 is involved in MMC-induced MN (wcp positive) less frequently than expected (*p < 0.01). Implementation of *χ* ^2^ test demonstrated that chromosomes 1, 9 and 16 are involved in BLM-induced MN as expected proportionally to their length (*p* > 0.05)

## Discussion

MN have been used widely as an easily evaluated indicator of chromosome damage; at the same time the chromosomal content of mutagen-induced MN is an important and yet not well-studied issue. In the present work, FISH was combined with CBMN test to characterize the content of MMC- and BLM-induced MN in human cells. Our objective was to analyze potential relationships between size, gene density and positioning of chromosomes in nucleus and MN formation.

MMC and BLM were chosen as the best-studied MN inducers in human lymphocytes [23] with different mechanisms of genotoxicity. MMC has been recognized as a classical DNA damaging agent, because of its monofunctional and bifunctional DNA alkylating activity and ability to cross-link the complementary strands of DNA [24]. The most frequent BLM-induced DNA lesions are single and double strand breaks and single apuinic/apyrimidinic sites. At the same time BLM is true radiomimetic compound, resembling almost completely the genetic effect of ionizing radiation [25].

The chromosomal content of MN was characterized by sequential application of cep and wcp probes to distinguish the presence of centric or acentric chromosomal fragments. The predominance of wcp compared to cep signals, especially in BLM-induced MN, indicates that besides clastogenic activity the mutagens applied also demonstrate aneugenic effect. Aneugenic activity was earlier shown for MMC [26] and BLM [23].

In our study the material of chromosomes 8, 15 and 20 was found in 1.51 %, 1.27 % and 0.55 % of *MMC-induced* MN versus 0 %, 1 % and 6 %, respectively, shown by Fauth et al. [15]. These differences can be explained by the higher number of scored MN (1,262 vs. 50−100) and lower dose of MMC (0.1 vs. 0.5 μg/ml) in our experiments, as well as by individual sensitivity of donors. We were unable to compare our data on BLM with the literature, since to our knowledge there are no publications on the chromosomal composition of *BLM-induced* human MN.

The analysis of chromosomes 8, 15 and 20 inclusion in *MMC-induced* MN demonstrated no correlation with chromosomal length/DNA-content. Negative correlation of cep-positive MN with gene density indicates that gene-rich chromosomes can be more secured from MMC aneugenic action. However, combined analysis of all by us analyzed twelve chromosomes (current: chromosomes 8, 15 and 20, and previous data: chromosomes 3, 4, 6, 7, 9, 16, 17, 18 and X [17]) demonstrated no correlation of number of cep- and wcp-positive MN with gene density and confirmed independence of micronucleation from chromosome size.

Chromosomes 1, 9 and 16 were studied here in *BLM-induced MN* as they are highly sensitive towards MMC [15, 17, 27, 28]. In contrast to MMC, material of these 3 chromosomes was detected in BLM-induced MN at a frequency proportional to their size relative to the entire genome. Correlations between cep- and wcp-positive MN and gene density in BLM-treated cells were not found.

The analysis of inclusion of each of now and earlier [17] studied chromosomes in *MMC-induced MN on the base of WCP-signals* revealed that material from most of them (3, 4, 6, 7, 8, 15, 17, 18, 20 and X) was found to be damaged less often than expected on the base of their size. Preferential involvement of chromosomes 9 and 16 in MN is in concordance with data from [15, 27–32] demonstrating that MMC induces undercondensation and breakage mainly in the pericentromeric heterochromatin blocks of chromosomes 1, 9 and 16.

In contrast to MMC results, material from each of chromosomes 1, 9 and 16 was involved in *BLM-induced MN* as expected at a frequency proportional to their size. Our data agreed with Promchainant [33] showing that the larger chromosomes are the more often they are involved in chromosomal aberrations in BLM-treated human leukocytes. Ellard et al. [34] revealed nonrandom distribution of BLM-induced damage in human chromosomes 1, 2 and 3; chromosome 1 was shown to be overrepresented in rearrangements. Difference with our data may be due to the ability of MN test to detect fewer aberrations than an analysis of chromosomes in metaphases [35]. Considering that BLM is known as a radiomimetic [25] we also compared our data with content of radiation-induced MN. Fimognari et al. [10] reported size-proportional inclusion of chromosomes 1, 7, 11, 14, 17 and 21 in MN *human lymphocytes*. Walker et al. [11] have shown that in *human skin fibroblasts* DNA in MN derived from smaller chromosomes (11 and 16) was observed as, and DNA from larger chromosomes (2 and 7) was incorporated in MN more frequently than expected, according to DNA-proportional distribution. The authors concluded that not all chromosomes in the human genome are equally susceptible to micronucleation. Balajee et al. [12] confirmed results of Walker et al. [11] on frequent involvement of larger chromosomes in radiation-induced MN. The authors consider that “frequencies of chromosomes micronucleation seem to correlate well with chromosome size because of a higher probability of double-strand breaks induction owing to spatial and temporal organization of chromatin in the interphase nuclei”. At the same time the involvement of chromosomes 13 and 19 in MN was observed at more than expected, suggesting that the formation of radiation-induced MN may not be always proportional to chromosome length. We consider our data on content of BLM-induced MN as preliminary, since they were obtained here only on the base of small set of chromosomes. Even though, our results do not contradict the literature data on the preferential damage of larger chromosomes in BLM- and in radiation-treated cells.

Different frequencies of involvement of chromosomes 1, 9 and 16 in MMC- and BLM-induced MN were detected. In MMC-induced MN chromosomes 1, 9 and 16 are included with frequencies 24 % [15], 31.6 % and 3.04 % [17], respectively. In the BLM-induced MN the frequency of chromosomes 1, 9 and 16 are 8.22 %, 4.69 % and 2.52 %, respectively. The correlation analysis revealed the involvement of these chromosomes in BLM-induced MN is size-dependent and in MMC-induced MN has size-independent character.

Although the nonrandom nature of interphase chromosome arrangement within the interphase nucleus is widely accepted, the relation of genome stability with nuclear organization remains mostly unclear [36]. It was demonstrated that relative positioning of chromosomes could be critical to determine chromosome damage [37–39]. However, it is unclear whether the spatial organization of chromosomes has functional consequences on MN formation. According to our data, the localization of chromosomes within interphase nucleus of human cells has no impact on their involvement in MMC- and BLM-induced MN. The involvement of whole chromosomes and their fragments in MN occurs during cell division and the results obtained can, therefore, be partially explained by drastic changes of interphase chromosome order during mitosis [40]. At the same time chromosomal breakage in the interphase may be dependent on chromosome position in interphase, determining the DNA accessibility to mutagens. Nevertheless, we were not able to reveal this relationship in the frame of our study.

## Conclusions

In this study, using cep and wcp FISH probes, we demonstrated that analyzed mutagens induce qualitatively different MN with regard to their chromosome composition. Different factors can contribute to the chromosome damage distribution and frequency of human chromosome material in MN.

Pooled analysis of involvement of chromosomes in MMC-induced MN revealed preferential micronucleation of chromosomes 9 and 16 and basically confirms well known high sensitivity of their heterochromatic blocks toward MMC. The involvement of chromosomes in BLM-induced MN positively correlates with chromosome length, thus the effect of radiomimetic BLM consistent with the data on composition of the radiation-induced MN.

Our results show the necessity of future investigations of contribution of different factors in distribution of mutagen-induced chromosome breakage and micronucleation in human cells. Last but not least this is important to use the correct mutagen for MN studies and to draw correct conclusions from the results.

## Methods

Peripheral blood was obtained from one female (25 years) and two male donors (25 and 29 years). The study was approved by the Ethical Committee of the Institute of Molecular Biology of National Academy of Sciences of RA (IRB # IORG 0002437), and informed consent was obtained from all three blood donors.

### CBMN technique

Heparinized whole blood was added to RPMI 1640 (Gibco) medium (1:10) containing 10 % fetal bovine serum (Biochrom), 1 % penicillin/streptomycin, and 10 μg/ml phytohemagglutinin (Biochrom). Whole blood was chosen as the most widely studied tissue in MN-tests, as it is more close to in vivo situation than using isolated lymphocytes [23]. The CBMN test was performed according to Fenech [1]. After 22 h cell cultures were treated with MMC (Sigma-Aldrich) or BLM sulfate (Sigma-Aldrich). The concentrations of MMC (0.1 μg/ml) and BLM (40 μg/ml) were chosen based upon previous dose–response experiments. Cytochalasin B (3 μg/ml; Sigma-Aldrich) was added after 44 h of incubation in order to block cytokinesis and obtain binucleated cells. In total, blood cultures were incubated for 72 h at 37 °C. Hypotonic treatment was performed for 3 min in cold 0.075 M KCl (Merk) at +4 °C. This procedure preserves the cytoplasm, which is required for the recognition of cell borders. Thus, MN can be assigned to their corresponding main nucleus. Fixation was done twice in ethanol/acetic acid (3:1). Slides were prepared by dropping and air drying. MN identification was done following DAPI (Sigma-Aldrich) staining in binucleated and mononucleated cells according to the criteria of Fenech [1].

### FISH technique

FISH was performed according to standard procedures [41]. In total, 1,262 MMC-induced MN from the three healthy donors were evaluated by a three-color-FISH probe set consisting of centromeric probes (cep) (Abbott/Vysis, Abbott GmbH & Co. KG, Wiesbaden, Germany) for chromosomes 8 (SpectrumGreen), 15 (SpectrumAqua) and CEP 20 (SpectrumOrange). 1,387 BLM-induced MN were hybridized and evaluated by a three-color-FISH probe set consisting of ceps (Abbott/Vysis) for chromosomes 9 (SpectrumAqua) and 16 (SpectrumGreen) as well as a probe for 1q12 (SpectrumOrange). The positions of MN on the slides were recorded for their further analysis by whole chromosome probes (WCP) prepared as in [42].

In the second round of hybridization, the same nuclei/MN in MMC-treated cells were hybridized with wcps for chromosomes 8 (SpectrumGreen), 15 (Cy5) and 20 (SpectrumOrange). Nuclei/MN in BLM-treated cells were hybridized with wcps for chromosomes 1 (SpectrumOrange), 9 (Cy5) and 16 (SpectrumGreen).

Image capturing and acquisition was processed with the Isis imaging system (MetaSystems, Altlussheim, Germany).

### Statistical analysis

Statistical analysis was performed by Pearson’s correlation and *χ*^2^ test using the statistical package SPSS version 19 (SPSS, Inc., an IBM Company, Chicago, IL).

## Abbreviations

BLM, bleomycin; CBMN, cytokinesis-block micronucleus; cep, centromeric probes; FISH, fluorescent *in situ* hybridization; MMC, mitomycin C; MN, micronuclei; SPSS, Statistical Package for the Social Sciences; wcp, whole-chromosome painting
